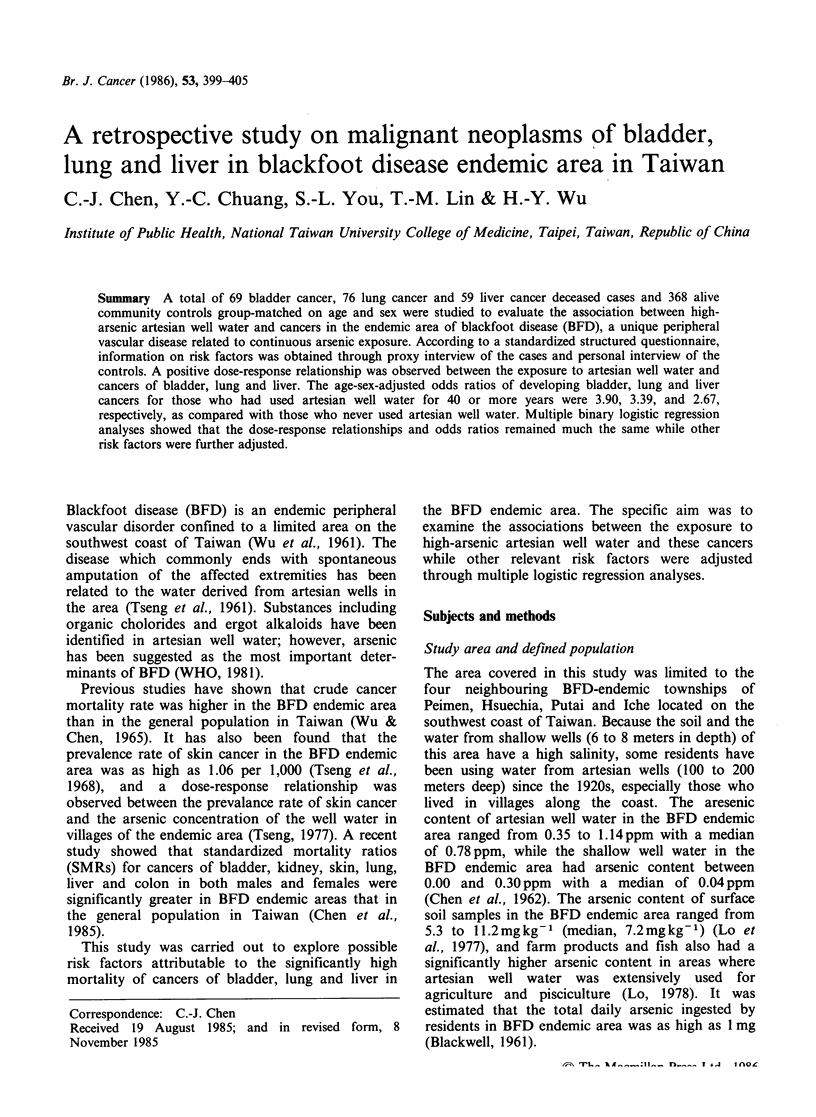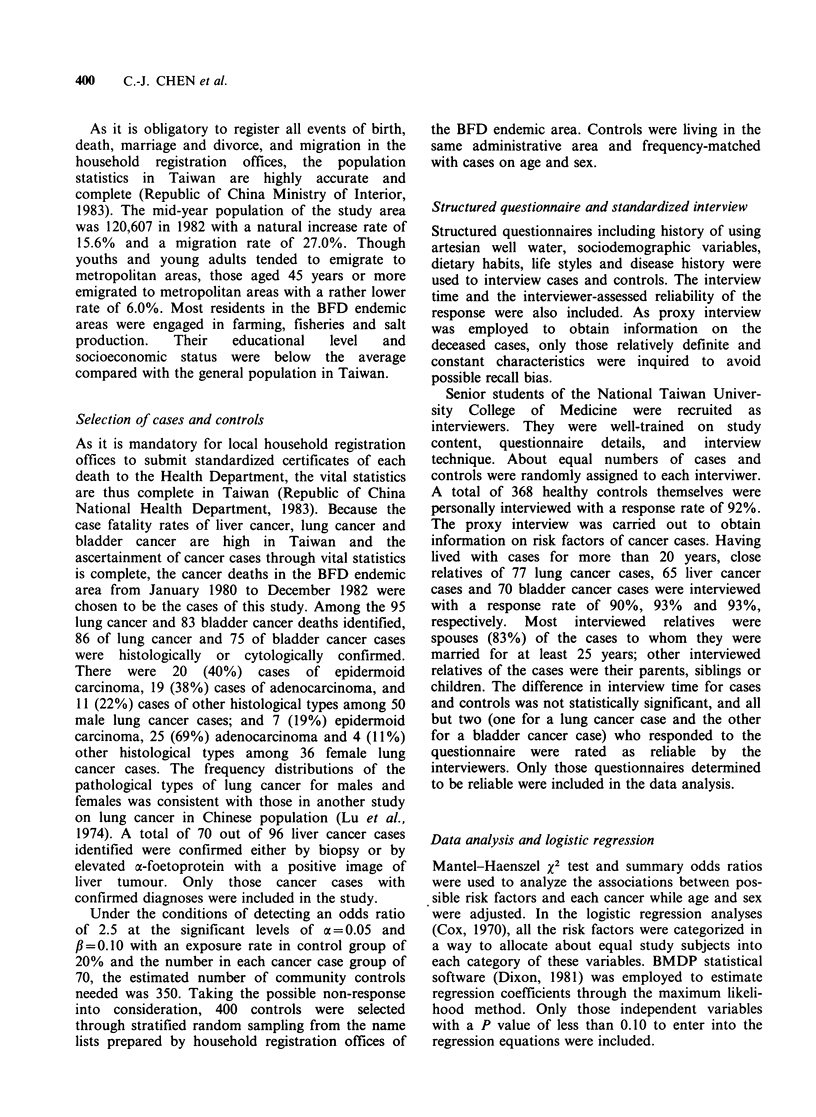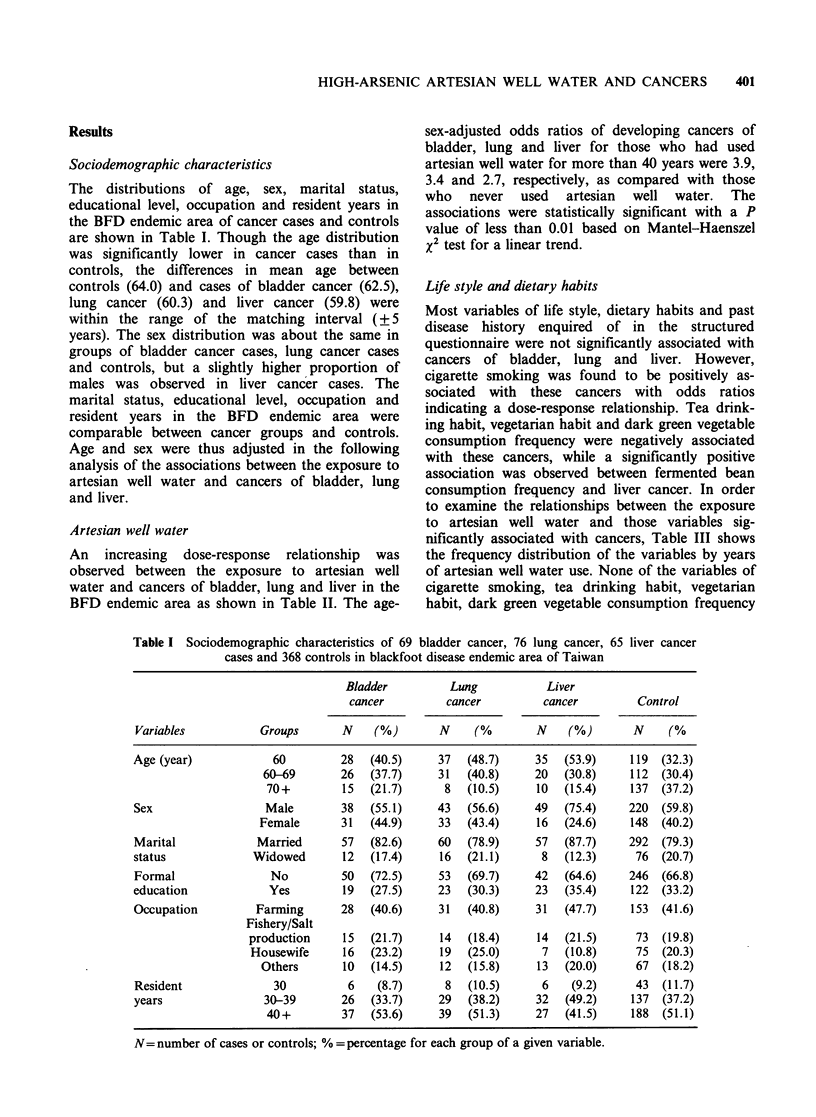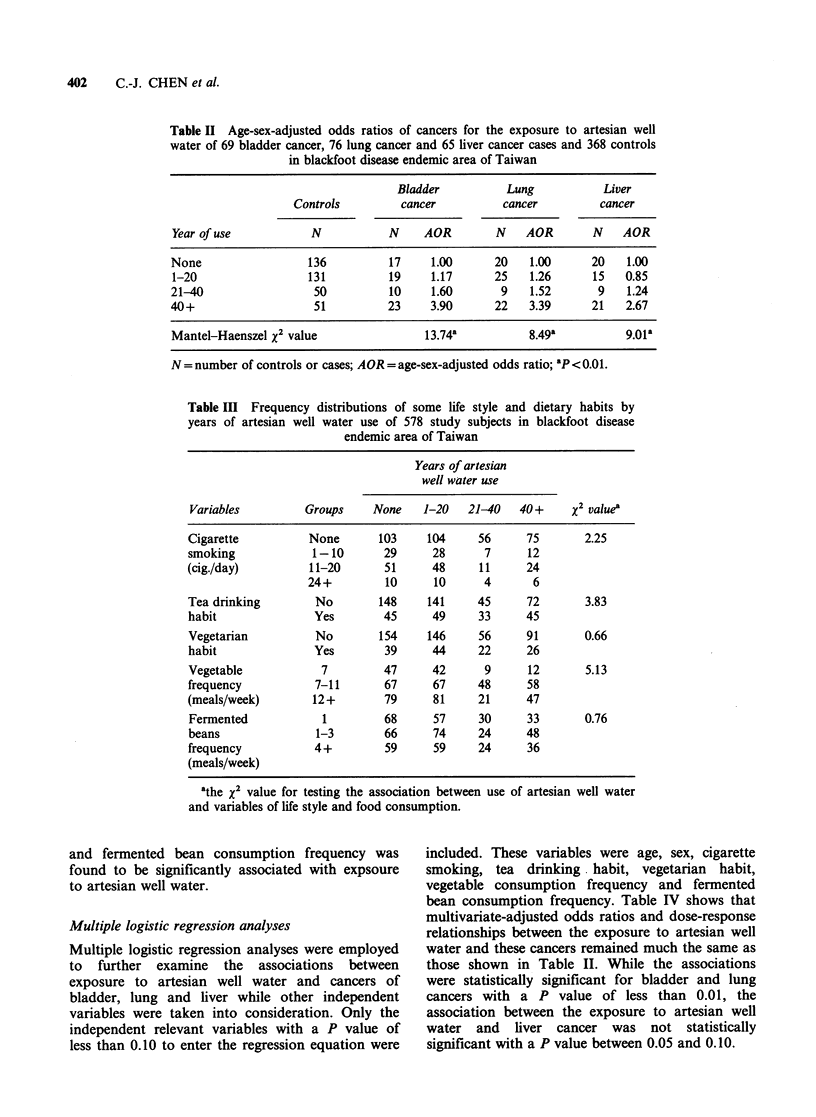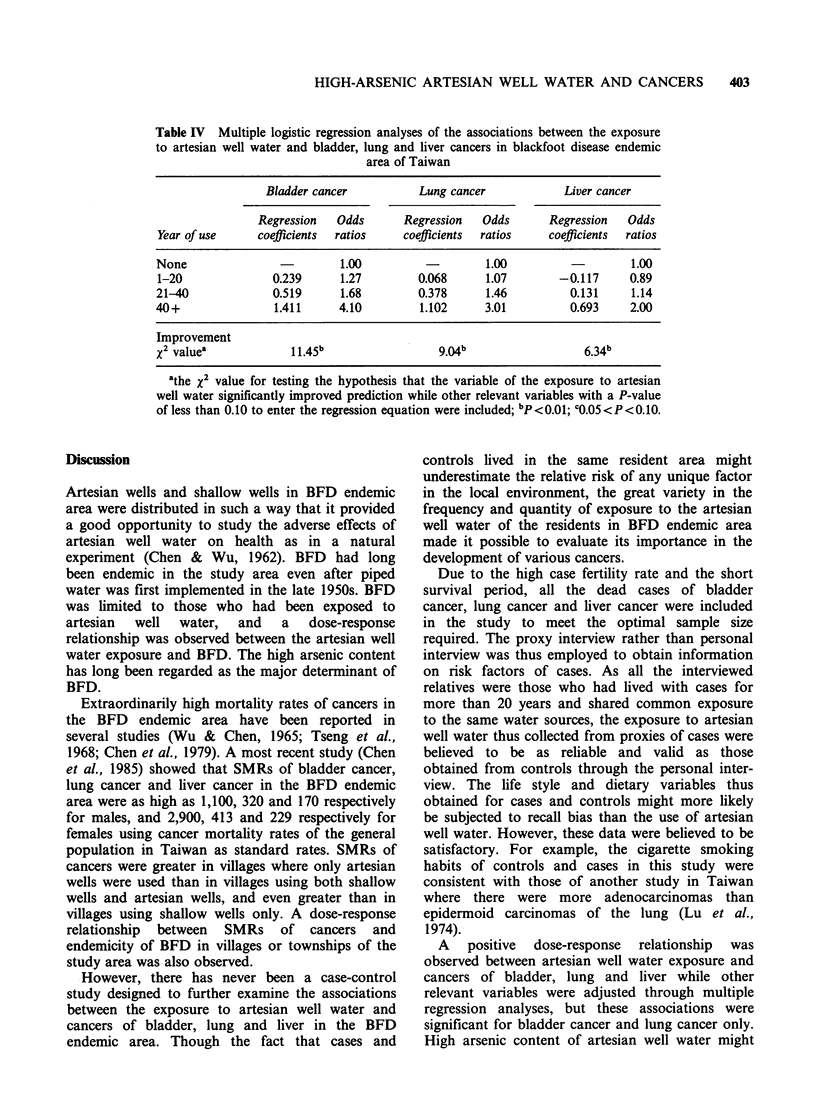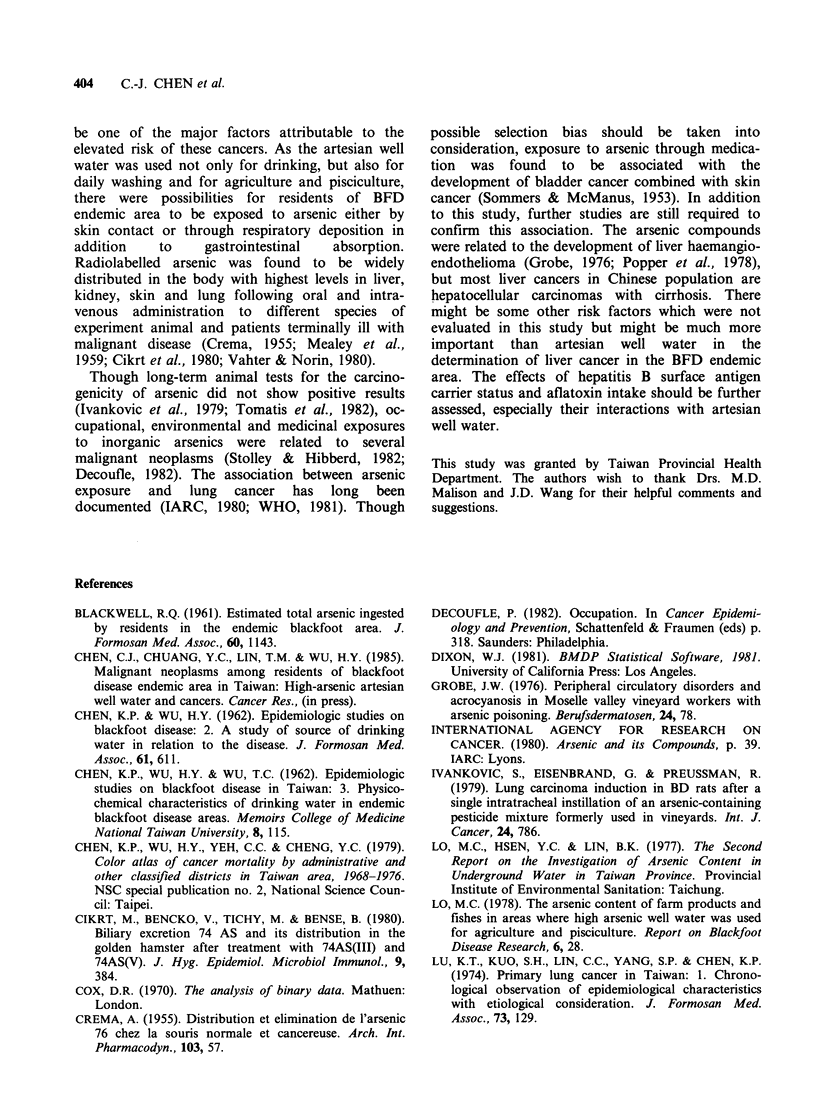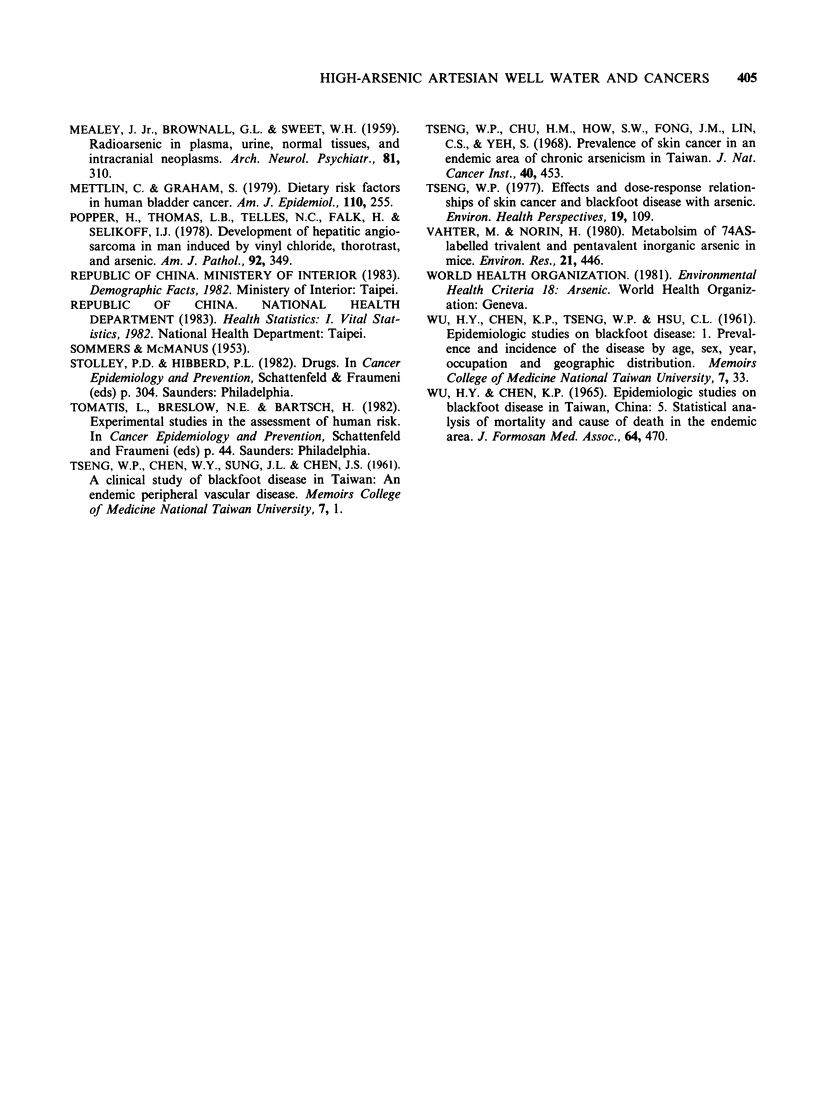# A retrospective study on malignant neoplasms of bladder, lung and liver in blackfoot disease endemic area in Taiwan.

**DOI:** 10.1038/bjc.1986.65

**Published:** 1986-03

**Authors:** C. J. Chen, Y. C. Chuang, S. L. You, T. M. Lin, H. Y. Wu

## Abstract

A total of 69 bladder cancer, 76 lung cancer and 59 liver cancer deceased cases and 368 alive community controls group-matched on age and sex were studied to evaluate the association between high-arsenic artesian well water and cancers in the endemic area of blackfoot disease (BFD), a unique peripheral vascular disease related to continuous arsenic exposure. According to a standardized structured questionnaire, information on risk factors was obtained through proxy interview of the cases and personal interview of the controls. A positive dose-response relationship was observed between the exposure to artesian well water and cancers of bladder, lung and liver. The age-sex-adjusted odds ratios of developing bladder, lung and liver cancers for those who had used artesian well water for 40 or more years were 3.90, 3.39, and 2.67, respectively, as compared with those who never used artesian well water. Multiple binary logistic regression analyses showed that the dose-response relationships and odds ratios remained much the same while other risk factors were further adjusted.


*Br. J. Cancer* (1986), **53**, 399–405

### A retrospective study on malignant neoplasms of bladder, lung and liver in blackfoot disease endemic area in Taiwan

C.-J. Chen, Y.-C. Chuang, S.-L. You, T.-M. Lin & H.-Y. Wu

*Institute of Public Health, National Taiwan University College of Medicine, Taipei, Taiwan, Republic of China*

**Summary** A total of 69 bladder cancer, 76 lung cancer and 59 liver cancer deceased cases and 368 alive community controls group-matched on age and sex were studied to evaluate the association between high-arsenic artesian well water and cancers in the endemic area of blackfoot disease (BFD), a unique peripheral vascular disease related to continuous arsenic exposure. According to a standardized structured questionnaire, information on risk factors was obtained through proxy interview of the cases and personal interview of the controls. A positive dose-response relationship was observed between the exposure to artesian well water and cancers of bladder, lung and liver. The age-sex-adjusted odds ratios of developing bladder, lung and liver cancers for those who had used artesian well water for 40 or more years were 3.90, 3.39, and 2.67, respectively, as compared with those who never used artesian well water. Multiple binary logistic regression analyses showed that the dose-response relationships and odds ratios remained much the same while other risk factors were further adjusted.

Blackfoot disease (BFD) is an endemic peripheral vascular disorder confined to a limited area on the southwest coast of Taiwan (Wu *et al.*, 1961). The disease which commonly ends with spontaneous amputation of the affected extremities has been related to the water derived from artesian wells in the area (Tseng *et al.*, 1961). Substances including organic cholorides and ergot alkaloids have been identified in artesian well water; however, arsenic has been suggested as the most important determinants of BFD (WHO, 1981).

Previous studies have shown that crude cancer mortality rate was higher in the BFD endemic area than in the general population in Taiwan (Wu & Chen, 1965). It has also been found that the prevalence rate of skin cancer in the BFD endemic area was as high as 1.06 per 1,000 (Tseng *et al.*, 1968), and a dose-response relationship was observed between the prevalance rate of skin cancer and the arsenic concentration of the well water in villages of the endemic area (Tseng, 1977). A recent study showed that standardized mortality ratios (SMRs) for cancers of bladder, kidney, skin, lung, liver and colon in both males and females were significantly greater in BFD endemic areas that in the general population in Taiwan (Chen *et al.*, 1985).

This study was carried out to explore possible risk factors attributable to the significantly high mortality of cancers of bladder, lung and liver in the BFD endemic area. The specific aim was to examine the associations between the exposure to high-arsenic artesian well water and these cancers while other relevant risk factors were adjusted through multiple logistic regression analyses.

### Subjects and methods

### *Study area and defined population*

The area covered in this study was limited to the four neighbouring BFD-endemic townships of Peimen, Hsuechia, Putai and Iche located on the southwest coast of Taiwan. Because the soil and the water from shallow wells (6 to 8 meters in depth) of this area have a high salinity, some residents have been using water from artesian wells (100 to 200 meters deep) since the 1920s, especially those who lived in villages along the coast. The aresenic content of artesian well water in the BFD endemic area ranged from 0.35 to 1.14 ppm with a median of 0.78 ppm, while the shallow well water in the BFD endemic area had arsenic content between 0.00 and 0.30 ppm with a median of 0.04 ppm (Chen *et al.*, 1962). The arsenic content of surface soil samples in the BFD endemic area ranged from 5.3 to 11.2 $mg\,kg^{-1}$ (median, 7.2 $mg\,kg^{-1}$) (Lo *et al.*, 1977), and farm products and fish also had a significantly higher arsenic content in areas where artesian well water was extensively used for agriculture and pisciculture (Lo, 1978). It was estimated that the total daily arsenic ingested by residents in BFD endemic area was as high as 1 mg (Blackwell, 1961).

Correspondence: C.-J. Chen
Received 19 August 1985; and in revised form, 8 November 1985

© The Macmillan Press Ltd., 1986

As it is obligatory to register all events of birth, death, marriage and divorce, and migration in the household registration offices, the population statistics in Taiwan are highly accurate and complete (Republic of China Ministry of Interior, 1983). The mid-year population of the study area was 120,607 in 1982 with a natural increase rate of 15.6% and a migration rate of 27.0%. Though youths and young adults tended to emigrate to metropolitan areas, those aged 45 years or more emigrated to metropolitan areas with a rather lower rate of 6.0%. Most residents in the BFD endemic areas were engaged in farming, fisheries and salt production. Their educational level and socioeconomic status were below the average compared with the general population in Taiwan.

*Selection of cases and controls*

As it is mandatory for local household registration offices to submit standardized certificates of each death to the Health Department, the vital statistics are thus complete in Taiwan (Republic of China National Health Department, 1983). Because the case fatality rates of liver cancer, lung cancer and bladder cancer are high in Taiwan and the ascertainment of cancer cases through vital statistics is complete, the cancer deaths in the BFD endemic area from January 1980 to December 1982 were chosen to be the cases of this study. Among the 95 lung cancer and 83 bladder cancer deaths identified, 86 of lung cancer and 75 of bladder cancer cases were histologically or cytologically confirmed. There were 20 (40%) cases of epidermoid carcinoma, 19 (38%) cases of adenocarcinoma, and 11 (22%) cases of other histological types among 50 male lung cancer cases; and 7 (19%) epidermoid carcinoma, 25 (69%) adenocarcinoma and 4 (11%) other histological types among 36 female lung cancer cases. The frequency distributions of the pathological types of lung cancer for males and females was consistent with those in another study on lung cancer in Chinese population (Lu *et al.*, 1974). A total of 70 out of 96 liver cancer cases identified were confirmed either by biopsy or by elevated α-foetoprotein with a positive image of liver tumour. Only those cancer cases with confirmed diagnoses were included in the study.

Under the conditions of detecting an odds ratio of 2.5 at the significant levels of $\alpha = 0.05$ and $\beta = 0.10$ with an exposure rate in control group of 20% and the number in each cancer case group of 70, the estimated number of community controls needed was 350. Taking the possible non-response into consideration, 400 controls were selected through stratified random sampling from the name lists prepared by household registration offices of the BFD endemic area. Controls were living in the same administrative area and frequency-matched with cases on age and sex.

*Structured questionnaire and standardized interview*

Structured questionnaires including history of using artesian well water, sociodemographic variables, dietary habits, life styles and disease history were used to interview cases and controls. The interview time and the interviewer-assessed reliability of the response were also included. As proxy interview was employed to obtain information on the deceased cases, only those relatively definite and constant characteristics were inquired to avoid possible recall bias.

Senior students of the National Taiwan University College of Medicine were recruited as interviewers. They were well-trained on study content, questionnaire details, and interview technique. About equal numbers of cases and controls were randomly assigned to each interviwer. A total of 368 healthy controls themselves were personally interviewed with a response rate of 92%. The proxy interview was carried out to obtain information on risk factors of cancer cases. Having lived with cases for more than 20 years, close relatives of 77 lung cancer cases, 65 liver cancer cases and 70 bladder cancer cases were interviewed with a response rate of 90%, 93% and 93%, respectively. Most interviewed relatives were spouses (83%) of the cases to whom they were married for at least 25 years; other interviewed relatives of the cases were their parents, siblings or children. The difference in interview time for cases and controls was not statistically significant, and all but two (one for a lung cancer case and the other for a bladder cancer case) who responded to the questionnaire were rated as reliable by the interviewers. Only those questionnaires determined to be reliable were included in the data analysis.

*Data analysis and logistic regression*

Mantel–Haenszel $\chi^2$ test and summary odds ratios were used to analyze the associations between possible risk factors and each cancer while age and sex were adjusted. In the logistic regression analyses (Cox, 1970), all the risk factors were categorized in a way to allocate about equal study subjects into each category of these variables. BMDP statistical software (Dixon, 1981) was employed to estimate regression coefficients through the maximum likelihood method. Only those independent variables with a $P$ value of less than 0.10 to enter into the regression equations were included.

### Results

### *Sociodemographic characteristics*

The distributions of age, sex, marital status, educational level, occupation and resident years in the BFD endemic area of cancer cases and controls are shown in Table I. Though the age distribution was significantly lower in cancer cases than in controls, the differences in mean age between controls (64.0) and cases of bladder cancer (62.5), lung cancer (60.3) and liver cancer (59.8) were within the range of the matching interval (±5 years). The sex distribution was about the same in groups of bladder cancer cases, lung cancer cases and controls, but a slightly higher proportion of males was observed in liver cancer cases. The marital status, educational level, occupation and resident years in the BFD endemic area were comparable between cancer groups and controls. Age and sex were thus adjusted in the following analysis of the associations between the exposure to artesian well water and cancers of bladder, lung and liver.

### *Artesian well water*

An increasing dose-response relationship was observed between the exposure to artesian well water and cancers of bladder, lung and liver in the BFD endemic area as shown in Table II. The age-sex-adjusted odds ratios of developing cancers of bladder, lung and liver for those who had used artesian well water for more than 40 years were 3.9, 3.4 and 2.7, respectively, as compared with those who never used artesian well water. The associations were statistically significant with a $P$ value of less than 0.01 based on Mantel–Haenszel $\chi^2$ test for a linear trend.

### *Life style and dietary habits*

Most variables of life style, dietary habits and past disease history enquired of in the structured questionnaire were not significantly associated with cancers of bladder, lung and liver. However, cigarette smoking was found to be positively associated with these cancers with odds ratios indicating a dose-response relationship. Tea drinking habit, vegetarian habit and dark green vegetable consumption frequency were negatively associated with these cancers, while a significantly positive association was observed between fermented bean consumption frequency and liver cancer. In order to examine the relationships between the exposure to artesian well water and those variables significantly associated with cancers, Table III shows the frequency distribution of the variables by years of artesian well water use. None of the variables of cigarette smoking, tea drinking habit, vegetarian habit, dark green vegetable consumption frequency

**Table I** Sociodemographic characteristics of 69 bladder cancer, 76 lung cancer, 65 liver cancer cases and 368 controls in blackfoot disease endemic area of Taiwan

| | | *Bladder cancer* | | *Lung cancer* | | *Liver cancer* | | *Control* | |
|---|---|---|---|---|---|---|---|---|---|
| *Variables* | *Groups* | *N* | *(%)* | *N* | *(%* | *N* | *(%)* | *N* | *(%* |
| Age (year) | 60 | 28 | (40.5) | 37 | (48.7) | 35 | (53.9) | 119 | (32.3) |
| | 60–69 | 26 | (37.7) | 31 | (40.8) | 20 | (30.8) | 112 | (30.4) |
| | 70+ | 15 | (21.7) | 8 | (10.5) | 10 | (15.4) | 137 | (37.2) |
| Sex | Male | 38 | (55.1) | 43 | (56.6) | 49 | (75.4) | 220 | (59.8) |
| | Female | 31 | (44.9) | 33 | (43.4) | 16 | (24.6) | 148 | (40.2) |
| Marital status | Married | 57 | (82.6) | 60 | (78.9) | 57 | (87.7) | 292 | (79.3) |
| | Widowed | 12 | (17.4) | 16 | (21.1) | 8 | (12.3) | 76 | (20.7) |
| Formal education | No | 50 | (72.5) | 53 | (69.7) | 42 | (64.6) | 246 | (66.8) |
| | Yes | 19 | (27.5) | 23 | (30.3) | 23 | (35.4) | 122 | (33.2) |
| Occupation | Farming | 28 | (40.6) | 31 | (40.8) | 31 | (47.7) | 153 | (41.6) |
| | Fishery/Salt production | 15 | (21.7) | 14 | (18.4) | 14 | (21.5) | 73 | (19.8) |
| | Housewife | 16 | (23.2) | 19 | (25.0) | 7 | (10.8) | 75 | (20.3) |
| | Others | 10 | (14.5) | 12 | (15.8) | 13 | (20.0) | 67 | (18.2) |
| Resident years | 30 | 6 | (8.7) | 8 | (10.5) | 6 | (9.2) | 43 | (11.7) |
| | 30–39 | 26 | (33.7) | 29 | (38.2) | 32 | (49.2) | 137 | (37.2) |
| | 40+ | 37 | (53.6) | 39 | (51.3) | 27 | (41.5) | 188 | (51.1) |

*N* = number of cases or controls; % = percentage for each group of a given variable.

**Table II** Age-sex-adjusted odds ratios of cancers for the exposure to artesian well water of 69 bladder cancer, 76 lung cancer and 65 liver cancer cases and 368 controls in blackfoot disease endemic area of Taiwan

| | *Controls* | *Bladder cancer* | | *Lung cancer* | | *Liver cancer* | |
|---|---|---|---|---|---|---|---|
| *Year of use* | *N* | *N* | *AOR* | *N* | *AOR* | *N* | *AOR* |
| None | 136 | 17 | 1.00 | 20 | 1.00 | 20 | 1.00 |
| 1–20 | 131 | 19 | 1.17 | 25 | 1.26 | 15 | 0.85 |
| 21–40 | 50 | 10 | 1.60 | 9 | 1.52 | 9 | 1.24 |
| 40+ | 51 | 23 | 3.90 | 22 | 3.39 | 21 | 2.67 |
| Mantel–Haenszel $\chi^2$ value | | | 13.74[a] | | 8.49[a] | | 9.01[a] |

$N$ = number of controls or cases; $AOR$ = age-sex-adjusted odds ratio; [a]$P<0.01$.

**Table III** Frequency distributions of some life style and dietary habits by years of artesian well water use of 578 study subjects in blackfoot disease endemic area of Taiwan

| | | *Years of artesian well water use* | | | | |
|---|---|---|---|---|---|---|
| *Variables* | *Groups* | *None* | *1–20* | *21–40* | *40+* | $\chi^2$ *value*[a] |
| Cigarette smoking (cig./day) | None | 103 | 104 | 56 | 75 | 2.25 |
| | 1–10 | 29 | 28 | 7 | 12 | |
| | 11–20 | 51 | 48 | 11 | 24 | |
| | 24+ | 10 | 10 | 4 | 6 | |
| Tea drinking habit | No | 148 | 141 | 45 | 72 | 3.83 |
| | Yes | 45 | 49 | 33 | 45 | |
| Vegetarian habit | No | 154 | 146 | 56 | 91 | 0.66 |
| | Yes | 39 | 44 | 22 | 26 | |
| Vegetable frequency (meals/week) | 7 | 47 | 42 | 9 | 12 | 5.13 |
| | 7–11 | 67 | 67 | 48 | 58 | |
| | 12+ | 79 | 81 | 21 | 47 | |
| Fermented beans frequency (meals/week) | 1 | 68 | 57 | 30 | 33 | 0.76 |
| | 1–3 | 66 | 74 | 24 | 48 | |
| | 4+ | 59 | 59 | 24 | 36 | |

[a]the $\chi^2$ value for testing the association between use of artesian well water and variables of life style and food consumption.

and fermented bean consumption frequency was found to be significantly associated with expsoure to artesian well water.

### *Multiple logistic regression analyses*

Multiple logistic regression analyses were employed to further examine the associations between exposure to artesian well water and cancers of bladder, lung and liver while other independent variables were taken into consideration. Only the independent relevant variables with a $P$ value of less than 0.10 to enter the regression equation were included. These variables were age, sex, cigarette smoking, tea drinking habit, vegetarian habit, vegetable consumption frequency and fermented bean consumption frequency. Table IV shows that multivariate-adjusted odds ratios and dose-response relationships between the exposure to artesian well water and these cancers remained much the same as those shown in Table II. While the associations were statistically significant for bladder and lung cancers with a $P$ value of less than 0.01, the association between the exposure to artesian well water and liver cancer was not statistically significant with a $P$ value between 0.05 and 0.10.

Table IV Multiple logistic regression analyses of the associations between the exposure to artesian well water and bladder, lung and liver cancers in blackfoot disease endemic area of Taiwan

| | *Bladder cancer* | | *Lung cancer* | | *Liver cancer* | |
|---|---|---|---|---|---|---|
| *Year of use* | *Regression coefficients* | *Odds ratios* | *Regression coefficients* | *Odds ratios* | *Regression coefficients* | *Odds ratios* |
| None | — | 1.00 | — | 1.00 | — | 1.00 |
| 1–20 | 0.239 | 1.27 | 0.068 | 1.07 | −0.117 | 0.89 |
| 21–40 | 0.519 | 1.68 | 0.378 | 1.46 | 0.131 | 1.14 |
| 40+ | 1.411 | 4.10 | 1.102 | 3.01 | 0.693 | 2.00 |
| Improvement $\chi^2$ value[a] | 11.45[b] | | 9.04[b] | | 6.34[b] | |

[a]the $\chi^2$ value for testing the hypothesis that the variable of the exposure to artesian well water significantly improved prediction while other relevant variables with a $P$-value of less than 0.10 to enter the regression equation were included; [b]$P<0.01$; [c]$0.05<P<0.10$.

### Discussion

Artesian wells and shallow wells in BFD endemic area were distributed in such a way that it provided a good opportunity to study the adverse effects of artesian well water on health as in a natural experiment (Chen & Wu, 1962). BFD had long been endemic in the study area even after piped water was first implemented in the late 1950s. BFD was limited to those who had been exposed to artesian well water, and a dose-response relationship was observed between the artesian well water exposure and BFD. The high arsenic content has long been regarded as the major determinant of BFD.

Extraordinarily high mortality rates of cancers in the BFD endemic area have been reported in several studies (Wu & Chen, 1965; Tseng *et al.*, 1968; Chen *et al.*, 1979). A most recent study (Chen *et al.*, 1985) showed that SMRs of bladder cancer, lung cancer and liver cancer in the BFD endemic area were as high as 1,100, 320 and 170 respectively for males, and 2,900, 413 and 229 respectively for females using cancer mortality rates of the general population in Taiwan as standard rates. SMRs of cancers were greater in villages where only artesian wells were used than in villages using both shallow wells and artesian wells, and even greater than in villages using shallow wells only. A dose-response relationship between SMRs of cancers and endemicity of BFD in villages or townships of the study area was also observed.

However, there has never been a case-control study designed to further examine the associations between the exposure to artesian well water and cancers of bladder, lung and liver in the BFD endemic area. Though the fact that cases and controls lived in the same resident area might underestimate the relative risk of any unique factor in the local environment, the great variety in the frequency and quantity of exposure to the artesian well water of the residents in BFD endemic area made it possible to evaluate its importance in the development of various cancers.

Due to the high case fertility rate and the short survival period, all the dead cases of bladder cancer, lung cancer and liver cancer were included in the study to meet the optimal sample size required. The proxy interview rather than personal interview was thus employed to obtain information on risk factors of cases. As all the interviewed relatives were those who had lived with cases for more than 20 years and shared common exposure to the same water sources, the exposure to artesian well water thus collected from proxies of cases were believed to be as reliable and valid as those obtained from controls through the personal interview. The life style and dietary variables thus obtained for cases and controls might more likely be subjected to recall bias than the use of artesian well water. However, these data were believed to be satisfactory. For example, the cigarette smoking habits of controls and cases in this study were consistent with those of another study in Taiwan where there were more adenocarcinomas than epidermoid carcinomas of the lung (Lu *et al.*, 1974).

A positive dose-response relationship was observed between artesian well water exposure and cancers of bladder, lung and liver while other relevant variables were adjusted through multiple regression analyses, but these associations were significant for bladder cancer and lung cancer only. High arsenic content of artesian well water might be one of the major factors attributable to the elevated risk of these cancers. As the artesian well water was used not only for drinking, but also for daily washing and for agriculture and pisciculture, there were possibilities for residents of BFD endemic area to be exposed to arsenic either by skin contact or through respiratory deposition in addition to gastrointestinal absorption. Radiolabelled arsenic was found to be widely distributed in the body with highest levels in liver, kidney, skin and lung following oral and intravenous administration to different species of experiment animal and patients terminally ill with malignant disease (Crema, 1955; Mealey et al., 1959; Cikrt et al., 1980; Vahter & Norin, 1980).

Though long-term animal tests for the carcinogenicity of arsenic did not show positive results (Ivankovic et al., 1979; Tomatis et al., 1982), occupational, environmental and medicinal exposures to inorganic arsenics were related to several malignant neoplasms (Stolley & Hibberd, 1982; Decoufle, 1982). The association between arsenic exposure and lung cancer has long been documented (IARC, 1980; WHO, 1981). Though possible selection bias should be taken into consideration, exposure to arsenic through medication was found to be associated with the development of bladder cancer combined with skin cancer (Sommers & McManus, 1953). In addition to this study, further studies are still required to confirm this association. The arsenic compounds were related to the development of liver haemangioendothelioma (Grobe, 1976; Popper et al., 1978), but most liver cancers in Chinese population are hepatocellular carcinomas with cirrhosis. There might be some other risk factors which were not evaluated in this study but might be much more important than artesian well water in the determination of liver cancer in the BFD endemic area. The effects of hepatitis B surface antigen carrier status and aflatoxin intake should be further assessed, especially their interactions with artesian well water.

This study was granted by Taiwan Provincial Health Department. The authors wish to thank Drs. M.D. Malison and J.D. Wang for their helpful comments and suggestions.

### References

BLACKWELL, R.Q. (1961). Estimated total arsenic ingested by residents in the endemic blackfoot area. *J. Formosan Med. Assoc.*, **60**, 1143.

CHEN, C.J., CHUANG, Y.C., LIN, T.M. & WU, H.Y. (1985). Malignant neoplasms among residents of blackfoot disease endemic area in Taiwan: High-arsenic artesian well water and cancers. *Cancer Res.*, (in press).

CHEN, K.P. & WU, H.Y. (1962). Epidemiologic studies on blackfoot disease: 2. A study of source of drinking water in relation to the disease. *J. Formosan Med. Assoc.*, **61**, 611.

CHEN, K.P., WU, H.Y. & WU, T.C. (1962). Epidemiologic studies on blackfoot disease in Taiwan: 3. Physicochemical characteristics of drinking water in endemic blackfoot disease areas. *Memoirs College of Medicine National Taiwan University*, **8**, 115.

CHEN, K.P., WU, H.Y., YEH, C.C. & CHENG, Y.C. (1979). *Color atlas of cancer mortality by administrative and other classified districts in Taiwan area, 1968–1976.* NSC special publication no. 2, National Science Council: Taipei.

CIKRT, M., BENCKO, V., TICHY, M. & BENSE, B. (1980). Biliary excretion 74 AS and its distribution in the golden hamster after treatment with 74AS(III) and 74AS(V). *J. Hyg. Epidemiol. Microbiol Immunol.*, **9**, 384.

COX, D.R. (1970). *The analysis of binary data.* Mathuen: London.

CREMA, A. (1955). Distribution et elimination de l'arsenic 76 chez la souris normale et cancereuse. *Arch. Int. Pharmacodyn.*, **103**, 57.

DECOUFLE, P. (1982). Occupation. In *Cancer Epidemiology and Prevention*, Schattenfeld & Fraumen (eds) p. 318. Saunders: Philadelphia.

DIXON, W.J. (1981). *BMDP Statistical Software, 1981.* University of California Press: Los Angeles.

GROBE, J.W. (1976). Peripheral circulatory disorders and acrocyanosis in Moselle valley vineyard workers with arsenic poisoning. *Berufsdermatosen*, **24**, 78.

INTERNATIONAL AGENCY FOR RESEARCH ON CANCER. (1980). *Arsenic and its Compounds*, p. 39. IARC: Lyons.

IVANKOVIC, S., EISENBRAND, G. & PREUSSMAN, R. (1979). Lung carcinoma induction in BD rats after a single intratracheal instillation of an arsenic-containing pesticide mixture formerly used in vineyards. *Int. J. Cancer*, **24**, 786.

LO, M.C., HSEN, Y.C. & LIN, B.K. (1977). *The Second Report on the Investigation of Arsenic Content in Underground Water in Taiwan Province.* Provincial Institute of Environmental Sanitation: Taichung.

LO, M.C. (1978). The arsenic content of farm products and fishes in areas where high arsenic well water was used for agriculture and pisciculture. *Report on Blackfoot Disease Research*, **6**, 28.

LU, K.T., KUO, S.H., LIN, C.C., YANG, S.P. & CHEN, K.P. (1974). Primary lung cancer in Taiwan: 1. Chronological observation of epidemiological characteristics with etiological consideration. *J. Formosan Med. Assoc.*, **73**, 129.

MEALEY, J. Jr., BROWNALL, G.L. & SWEET, W.H. (1959). Radioarsenic in plasma, urine, normal tissues, and intracranial neoplasms. *Arch. Neurol. Psychiatr.*, **81**, 310.

METTLIN, C. & GRAHAM, S. (1979). Dietary risk factors in human bladder cancer. *Am. J. Epidemiol.*, **110**, 255.

POPPER, H., THOMAS, L.B., TELLES, N.C., FALK, H. & SELIKOFF, I.J. (1978). Development of hepatitic angiosarcoma in man induced by vinyl chloride, thorotrast, and arsenic. *Am. J. Pathol.*, **92**, 349.

REPUBLIC OF CHINA. MINISTERY OF INTERIOR (1983). *Demographic Facts, 1982.* Ministery of Interior: Taipei.

REPUBLIC OF CHINA. NATIONAL HEALTH DEPARTMENT (1983). *Health Statistics: I. Vital Statistics, 1982.* National Health Department: Taipei.

SOMMERS & McMANUS (1953).

STOLLEY, P.D. & HIBBERD, P.L. (1982). Drugs. In *Cancer Epidemiology and Prevention,* Schattenfeld & Fraumeni (eds) p. 304. Saunders: Philadelphia.

TOMATIS, L., BRESLOW, N.E. & BARTSCH, H. (1982). Experimental studies in the assessment of human risk. In *Cancer Epidemiology and Prevention,* Schattenfeld and Fraumeni (eds) p. 44. Saunders: Philadelphia.

TSENG, W.P., CHEN, W.Y., SUNG, J.L. & CHEN, J.S. (1961). A clinical study of blackfoot disease in Taiwan: An endemic peripheral vascular disease. *Memoirs College of Medicine National Taiwan University,* **7**, 1.

TSENG, W.P., CHU, H.M., HOW, S.W., FONG, J.M., LIN, C.S., & YEH, S. (1968). Prevalence of skin cancer in an endemic area of chronic arsenicism in Taiwan. *J. Nat. Cancer Inst.*, **40**, 453.

TSENG, W.P. (1977). Effects and dose-response relationships of skin cancer and blackfoot disease with arsenic. *Environ. Health Perspectives,* **19**, 109.

VAHTER, M. & NORIN, H. (1980). Metabolsim of 74AS-labelled trivalent and pentavalent inorganic arsenic in mice. *Environ. Res.*, **21**, 446.

WORLD HEALTH ORGANIZATION. (1981). *Environmental Health Criteria 18: Arsenic.* World Health Organization: Geneva.

WU, H.Y., CHEN, K.P., TSENG, W.P. & HSU, C.L. (1961). Epidemiologic studies on blackfoot disease: 1. Prevalence and incidence of the disease by age, sex, year, occupation and geographic distribution. *Memoirs College of Medicine National Taiwan University,* **7**, 33.

WU, H.Y. & CHEN, K.P. (1965). Epidemiologic studies on blackfoot disease in Taiwan, China: 5. Statistical analysis of mortality and cause of death in the endemic area. *J. Formosan Med. Assoc.*, **64**, 470.