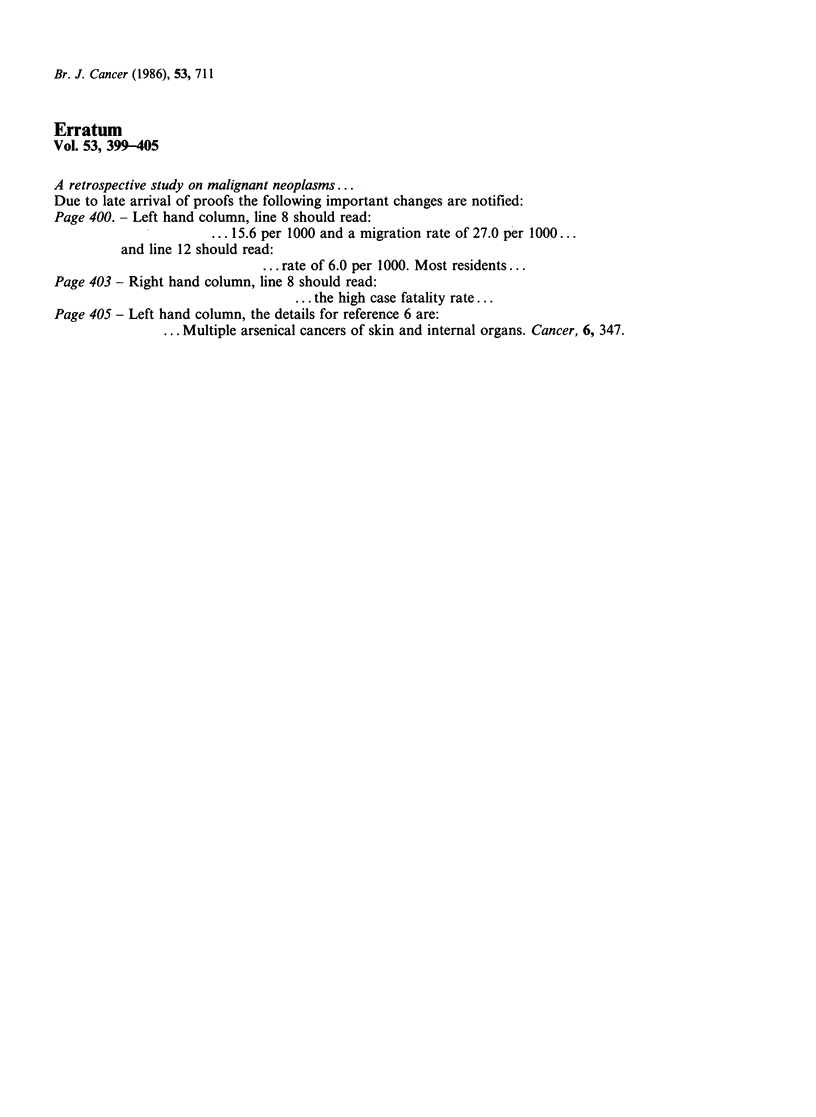# Erratum

**Published:** 1986-05

**Authors:** 


*Br. J. Cancer* (1986), **53**, 711

### Erratum
**Vol. 53, 399–405**

*A retrospective study on malignant neoplasms...*

Due to late arrival of proofs the following important changes are notified:

*Page 400*. – Left hand column, line 8 should read:

...15.6 per 1000 and a migration rate of 27.0 per 1000...

and line 12 should read:

...rate of 6.0 per 1000. Most residents...

*Page 403* – Right hand column, line 8 should read:

...the high case fatality rate...

*Page 405* – Left hand column, the details for reference 6 are:

...Multiple arsenical cancers of skin and internal organs. *Cancer,* **6,** 347.